# Fatal aluminium phosphide poisoning due to myocardial depression refractory to high dose inotropic support and intra-aortic balloon counterpulsation

**DOI:** 10.4103/0972-5229.40949

**Published:** 2008

**Authors:** J. Chacko, C. Shivaprasad

**Affiliations:** **From:** Department of Critical Care, Narayana Hrudayalaya Hospital, Bangalore, India

**Keywords:** Aluminium phospide, intraaortic balloon counterpulsation, myocardial depression

## Abstract

We report a case of aluminium phosphide poisioning that presented to us with refractory myocardial depression. This patient developed worsening circulatory failure that did not respond to inotropic or vasopressor support and intra-aortic balloon counterpulsation. She went on to develop progressive metabolic acidosis, renal failure and died within 48 hours of admission to the intensive care unit.

## Introduction

A 25-year-old lady was brought to the Emergency Department of our hospital half an hour after having consumed 10 tablets of aluminium phosphide (ALP) 50%. At the time of presentation, she was nauseated and had several episodes of vomiting. Her heart rate was 120/min, BP was 110/75 mmHg, she was breathing at a rate of 20 min, with oxygen saturations around 98-99% on room air. Initial blood gas analysis revealed significant metabolic acidosis with a pH: 7.33, PaCO2: of 23.3, HCO3: 12.1 and base excess: −13.8. A gastric lavage was given and she was transferred to the intensive care unit where she was put on invasive blood pressure and central venous pressure monitoring. Her initial liver function tests, blood urea nitrogen, creatinine, creatinine kinase, troponin-I and magnesium levels were within the normal range. Four hours after admission, she developed hypotension (systolic pressures less than 90 mmHg and MAP less than 60 mmHg). Fluid boluses up to 1.5 L of normal saline were given to raise the central venous pressure to 16; however her blood pressure continued to be low. She was started on a dobutamine infusion at 5 mcg/kg/min and a bicarbonate infusion at 20 mmols/hr. She was also given intravenous magnesium sulphate to keep the serum magnesium levels around the upper limit of normal. With this management she remained hemodynamically stable and maintained a urine output of 75 to 100 mls/hr. Her metabolic acidosis persisted, though it was improving with ongoing infusion of bicarbonate. The following morning (about 14 h after admission), she became more tachycardic, with heart rates up to 160/min. She was also becoming increasingly tachypnoeic and her metabolic acidosis persisted. She was intubated and put on mechanical ventilation at this time - which led to further drop in blood pressures, with high central venous pressures. She was started on adrenaline infusion at this time, initially at 0.05 mcg/kg/mt. Further magnesium supplements were given to raise her magnesium level to the upper limit of normal. She was initiated on continuous veno-venous hemodiafiltration (CVVHD) at this time in view of the persisting metabolic acidosis. After initiation of CVVHD, her acidosis improved, though she continued to remain tachycardic and hypotensive in spite of increasing doses of adrenaline. An echocardiogram revealed severe impairment of left ventricular function with an ejection fraction of 30% on high level of inotropic support with dobutamine and adrenaline. Her troponin-I level had increased at this point in time to 12.7 ng/ml (normal level: 0.0 to 0.4 ng/ml). In view of her worsening cardiac function in spite of increasing doses of inotropic support, an intraaortic balloon pump was inserted to support LV function. Augmentation was commenced at 1:2 support considering her significant tachycardia. The IABP had no apparent effect on her hemodynamic status even after several hours of support. She continued to be tachycardic, hypotensive with high central venous pressures and persisting metabolic acidosis in spite of ongoing CVVHD. Despite continued supportive measures, she never showed any improvement and died about 48h after admission to the intensive care unit.

## Discussion

Aluminium phosphide (ALP) is used as a rodenticide and is a common agent used in suicidal attempts in northern India. Refractory myocardial depression from ALP toxicity is not uncommon and carries a mortality of up to 77%.[1,2] ALP releases phosphine gas when it comes in to contact with moisture, which is then absorbed rapidly through the gastrointestinal tract, lungs or the skin. Phosphine causes non-competitive inhibition of mitochondrial cytochrome oxidase.[3] This has been suggested to inhibit myocardial cellular metabolism and necrosis of the cardiac tissue resulting in the release of reactive oxygen intermediates.[4] Hypomagnesaemia has been known to cause arrhythmias in ALP poisoning and magnesium supplementation has been suggested as a therapeutic option.[2] However, supplemental magnesium had no discernible effect on our patient. Intraaortic ballon counterpulsation (IABP) has been used successfully as a temporizing measure in toxicity due to other myocardial depressant agents.[5–7] We tried IABP to try to mechanically support the heart. Even after several hours of optimal augmentation, this had no positive effect on our patient's hemodynamic status. We did consider the option of using cardiopulmonary bypass (CPB) as a support measure, however, the fairly rapid terminal phase of her illness did not permit us to do so.

We present a case of refractory myocardial depression from ALP toxicity. In spite of inotropic support with high doses of dobutamine and adrenaline, our patient went on a rapid downhill course with worsening tachycardia, hypotension, metabolic acidosis and very high troponin-I levels, possibly due to extensive myocardial necrosis. There was no improvement in her hemodynamic status with intraaortic balloon counterpulsation. To our knowledge, there is no other report in literature of IABP use to try to support the myocardium in ALP poisoning. The possible benefit of CPB would need to be explored in refractory myocardial depression due to ALP.

## References

[1] Bogle RG, Theron P, Brooks P, Dargan PI, Redhead J (2006). Aluminium phosphide poisoning. Emerg Med J.

[2] Chugh SN, Dushyant, Ram S, Arora B, Malhotra KC (1991). Incidence and outcome of aluminium phosphide poisoning in a hospital study. Indian J Med Res.

[3] Sudakin DL (2005). Occupational exposure to aluminium phosphide and phosphine gas? A suspected case report and review of the literature. Hum Exp Toxicol.

[4] Lall SB, Sinha K, Mittra S, Seth SD (1997). An experimental study on cardiotoxicity of aluminium phosphide. Indian J Exp Biol.

[5] Frierson J, Bailly D, Shultz T, Sund S, Dimas A (1991). Refractory cardiogenic shock and complete heart block after unsuspected verapamil-SR and atenolol overdose. Clin Cardiol.

[6] Lane AS, Woodward AC, Goldman MR (1987). Massive propranolol overdose poorly responsive to pharmacologic therapy: Use of the intra-aortic balloon pump. Ann Emerg Med.

[7] Timperley J, Mitchell AR, Brown PD, West NE (2005). Flecainide overdose-support using an intra-aortic balloon pump. BMC Emerg Med.

